# Lutein Prevents Liver Injury and Intestinal Barrier Dysfunction in Rats Subjected to Chronic Alcohol Intake

**DOI:** 10.3390/nu15051229

**Published:** 2023-02-28

**Authors:** Suli Zhao, Yebing Zhang, Haoyue Ding, Shouna Hu, Xiaoqing Wu, Aiguo Ma, Yan Ma

**Affiliations:** 1 Institute of Nutrition and Health, School of Public Health, Qingdao University, Qingdao 266071, China; 2 Department of Nutrition and Food Hygiene, School of Public Health, Soochow University, Suzhou 215127, China

**Keywords:** lutein, alcohol, alcoholic liver injury, intestinal barrier, oxidative stress

## Abstract

Chronic alcohol intake can affect both liver and intestinal barrier function. The goal of this investigation was to evaluate the function and mechanism of lutein administration on the chronic ethanol-induced liver and intestinal barrier damage in rats. During the 14-week experimental cycle, seventy rats were randomly divided into seven groups, with 10 rats in each group: a normal control group (Co), a control group of lutein interventions (24 mg/kg/day), an ethanol model group (Et, 8–12 mL/kg/day of 56% (v/v) ethanol), three intervention groups with lutein (12, 24 and 48 mg/kg/day) and a positive control group (DG). The results showed that liver index, ALT, AST and TG levels were increased, and SOD and GSH-Px levels were reduced in the Et group. Furthermore, alcohol intake over a long time increased the level of pro-inflammatory cytokines TNF-α and IL-1β, disrupted the intestinal barrier, and stimulated the release of LPS, causing further liver injury. In contrast, lutein interventions prevented alcohol-induced alterations in liver tissue, oxidative stress and inflammation. In addition, the protein expression of Claudin-1 and Occludin in ileal tissues was upregulated by lutein intervention. In conclusion, lutein can improve chronic alcoholic liver injury and intestinal barrier dysfunction in rats.

## 1. Introduction

Alcoholic liver disease (ALD) refers to a group of liver pathological changes that include hepatic steatosis, alcoholic hepatitis and alcoholic liver fibrosis [1]. One of the primary causes of the condition is the chronic excessive intake of alcohol [2]. Specifically, chronic alcohol exposure can induce the enhanced activity of cytochrome P450 2E1 (CYP2E1) in the liver, contributing to the buildup of excessive reactive oxygen species (ROS) in the body, which can cause hepatic oxidative stress, dysregulation of lipid metabolism and inflammation [3,4]. Furthermore, chronic alcohol intake can also lead to oxidative damage in the gut [5], resulting in high intestinal permeability and ecological dysbiosis of the microbiota, causing endotoxemia in the organism and leading to further liver damage [6]. In addition to abstinence from alcohol and hepatoprotective drug therapy, some studies have found that supplementation with antioxidant and/or anti-inflammatory dietary supplements, such as carotenoids, vitamins, curcumin and probiotics, can be beneficial remedies for alcoholic liver disease [7].

Lutein is a carotenoid with antioxidant and anti-inflammatory properties [8] which is abundant in egg yolk and dark green leafy vegetables [9,10] and is generally regarded as a safe (GRAS) molecule [11]. By supplementing with lutein, aberrant lipid metabolism in the liver may be effectively resolved [12], and arsenic-induced oxidative stress damage in the liver can be alleviated [13]. Furthermore, lutein decreases LPS-induced mortality in mice via modifying NF-κB-mediated inflammatory pathways [14]. Early studies have found that supplementation with lutein might improve the levels of antioxidant enzymes in rats and thus play a protective role against long-term ethanol intake-induced liver damage, but the specific mechanism is still unclear [15]. In vitro studies have shown that lutein reduces intestinal tight junction opening and barrier impairment [16], and population studies have revealed that blood lutein levels in children are linked with intestinal barrier dysfunction even after controlling for seasonal variables [17]. It has been found that alcohol intake might disrupt the intestinal barrier and cause liver damage in the body [2], but it is uncertain what role lutein plays in chronic alcohol intake-induced intestinal barrier disruption and liver damage.

The goal of this study was to assess the role and mechanism of lutein in liver injury and intestinal barrier dysfunction caused by chronic alcohol consumption, as well as the effect of lutein on intestinal microbiota in rats.

## 2. Materials and Methods

### 2.1. Materials

The lutein (C_40_H_56_O_2_, HPLC 80%) was provided by Shanghai Yuanye Biotechnology Co. (Shanghai, China). The alcohol (56% (v/v) ethanol) was provided by Beijing Red Star Co. (Beijing, China). Diammonium glycyrrhizinate, which possesses excellent anti-inflammatory, immunomodulatory and liver function enhancement properties, was supplied by Lianyungang Zhengda Tianqing Pharmaceutical Group Co. (Lianyungang, China) [18]. The corn oil was supplied by Yihai Kerry Arawana Holdings Co., Ltd. (Shanghai, China). All remaining reagents utilized in this investigation were of analytical or HPLC purity.

### 2.2. Experimental Models and Treatments

Seventy male Wistar rats (weight 140–160 g, 6 weeks old) were acquired from SPF (Beijing, China) Biotechnology Co. Rats were acclimatized for two weeks in a 12 h light/dark cycle at the proper temperature (22–25 °C) and humidity (50–60%). Food and water were available at all times. The International Guide for the Care of Laboratory Animals was followed, and all animal experiments in this work were authorized by the Qingdao University Experimental Animal Welfare Ethics Committee (No. 20210315Wistar7720210706104).

At the end of the acclimatization feeding, rats were randomly allocated to a normal control group (Co, *n* = 10), a control group of medium-dose lutein interventions (CoLU, 24 mg per kg BW, *n* = 10), an ethanol model group (Et, *n* =10), an intervention group with low-dose lutein (LLU, 12 mg per kg BW, *n* = 10), an intervention group with medium-dose lutein (MLU, 24 mg per kg BW, *n* = 10), an intervention group with high-dose lutein (HLU, 48 mg per kg BW, *n* = 10), and a positive control group (DG, diammonium glycyrrhizinate, 200 mg per kg BW, *n* = 10). The gavage dose of lutein was chosen with reference to previous studies [12,19]. Lutein dissolved in corn oil was given to each intervention group every day, while the other groups were given equal amounts of corn oil for 14 weeks. From the third week, the DG group was given diammonium glycyrrhizinate, and all groups except the two control groups were given alcohol (8 mL per kg BW of 56% (v/v) ethanol (3.54 g/kg BW ethanol)) orally for two weeks to acclimatize them to alcohol consumption, followed by 12 mL per kg BW of 56% (v/v) ethanol (5.30 g/kg BW ethanol)) orally for the next 10 weeks by gavage 6 h after administration of lutein or diammonium glycyrrhizinate. After the last gavage operation, fasting was performed for 12 h. Rats’ abdominal aortas were sampled for blood for subsequent index testing. Samples of rat liver tissue, small intestinal tissue, and cecum contents were also quickly removed for other experiments.

### 2.3. Histopathological Analysis of Liver and Small Intestine

Fresh liver tissue was dissected quickly, and the liver index was determined as follows: liver index (%) = liver weight (g)/body weight (g) × 100%. The small intestine was dissected, and the length of the duodenum, jejunum and ileum was measured. Following that, small amounts of liver tissue and ileum tissue were put in paraformaldehyde (4%) solution and embedded in paraffin, and the paraffin blocks were cut into 5 μm thick sections using the frozen section technique for the preparation of hematoxylin and eosin (H&E) staining. A histopathological examination of the liver and the ileum was performed using a light microscope (BX60, Olympus, Tokyo, Japan). Liver pathology was scored by pathologists in a blinded manner according to previously published scoring criteria [20], as detailed in Table 1. In addition, small intestinal villus length and intestinal crypt depth in the field of view of ileal sections were measured under a microscope for each group.

### 2.4. Biochemical Index Measurement

To collect serum for further testing, blood was centrifuged at 3000 rpm for 10 min at low temperatures followed by the determination of alanine aminotransferase (ALT), aspartate aminotransferase (AST), triglyceride (TG) and total cholesterol (TC) levels in serum using appropriate kits (Nanjing Jiancheng Institute of Biological Engineering, Nanjing, China). The level of Gamma-glutamyl transferase (GGT) in serum was determined using a fully automated biochemical analyzer (AU5400, Beckman, Los Angeles, CA, USA). Under low-temperature settings, liver tissue homogenates were produced by centrifugation with pre-cooled saline at 12,000 rpm for 10 min. The levels of glutathione peroxidase (GSH-Px), catalase (CAT), malondialdehyde (MDA) in the liver, and superoxide dismutase (SOD) and total antioxidant capacity (T-AOC) in serum were measured using appropriate kits (Nanjing Jiancheng Institute of Biological Engineering, Nanjing, China). The enzyme-linked immunosorbent test kits (Wuhan BOSTER Bioengineering Co., Ltd., Beijing, China) were employed to assess the amounts of interleukin 1β (IL-1β) and tumor necrosis factor α (TNF-α) in the serum. In addition, serum levels of D-lactate (D-LA), intestinal fatty acid binding protein (FABP2), as well as lipopolysaccharide-binding protein (LBP), were assessed using enzyme-linked immunosorbent test kits (Nanjing Jiancheng Institute of Biological Engineering, Nanjing, China). The level of lipopolysaccharide (LPS) in serum was also determined using enzyme-linked immunosorbent assay kits (Xiamen Huijia Biotechnology Co., Ltd., Xiamen, China).

### 2.5. Western Blot

Cytoplasmic or nuclear proteins were extracted from liver and ileum tissues according to the kit instructions (Jiangsu KeyGEN BioTECH Co., Ltd., Nanjing, China); the specific steps are described in the Supplementary Materials. The concentrations of protein extracts were measured using appropriate kits (Beyotime, Zhenjiang, China). Subsequently, the protein extracts were separated using 10% SDS-PAGE gels before being transferred to polyvinylidene difluoride (PVDF) membranes, which were subsequently bathed in 10% skim milk, then incubated with primary antibodies CYP2E1 (1:1000), NF-κB (1:10,000) (Abcam, Cambridge, UK), Nrf2 (1:3000) (Affinity, Nanjing, China), HO-1 (1:3000) (Abmart, Shanghai, China), MyD88 (1:1000) (Boster, Wuhan, China), TLR4 (1:1000), IκB-α (1:1000) (CST, Boston, MA, USA), Occludin (1:1000), Claudin-1 (1:1000), ZO-1 (1:1000) (Proteintech, Wuhan, China), β-actin (1:10,000) (Abways, Shanghai, China), or Lamin B1 (1:10,000) (Bioworld, Minneapolis, MN, USA) overnight at 4 °C. The membranes were then rinsed three times for ten minutes each with 1 × TBST. The washed primary antibody response membranes were incubated for one hour with a matching secondary antibody (Bioeasy, Beijing, China) at room temperature, protected from light, then washed and exposed to appropriate developing reagents. β-actin and Lamin B1 were utilized as cytoplasmic and nuclear protein reference proteins, respectively.

### 2.6. Real-Time Quantitative Polymerase Chain Reaction

Total RNA was extracted from rat liver and ileum using TRIZOL per the instructions (Tiangen Biochemical Technology (Beijing) Co., Beijing, China). The isolated RNA was reverse transcribed to cDNA and subsequently analyzed by real-time fluorescent quantitative polymerase chain reaction (PCR) with gene-specific primers, namely, *ADH1*, *ALDH2*, *Claudin*-*1*, *Occludin*, *ZO-1* and *β-actin*. Amplification was performed as described in [21]. The sequences of the gene primers are shown in Table 2.

### 2.7. Determination of Microbiota in Cecal Contents

Since cecum bacteria content was abundant and sufficient, fecal samples could be obtained. Six cecum content samples were randomly selected from each group and 16S rDNA gene sequencing was performed by Beijing Biomarker Technologies Co (Biomarker Technologies Co., Ltd., Beijing, China). The flora in the cecum content was extracted according to the method in [22]. The V3–V4 region of the 16S rRNA gene was amplified using PCR (universal primers, forward (5′–3′): ACTCCTACGGGAGGCAGCA and reverse (5′–3′): GGACTACHVGGGTWTCTAAT) to obtain the amplified product, which was subsequently sequenced using the Illumina NovaSeq 6000 platform. The valid data (non-chimeric reads) obtained were subjected to α-diversity and β-diversity analysis using QIIME2 2020.6 software. The α-diversity and β-diversity of each group were analyzed by the Wilcoxon rank sum test. Line Discriminant Analysis (LDA) Effect Size (LEfSe) was used to find biomarkers with statistical differences among groups from the gate level to the species level. Subsequently, the Kruskal–Wallis test was used to further search for differential bacteria at the genus level, and the results were corrected using the false discovery rate based on the Benjamini–Hochberg method (BH-FDR). The raw data have been uploaded to the NCBI database with the accession number PRJNA879916.

### 2.8. Statistical Analysis

Except for the intestinal microbiota, all data are shown as mean ± SEM, and statistical differences among groups were examined using one-way analysis of variance (ANOVA), with multiple comparisons using the least significant difference (LSD). The Kruskal–Wallis test in the nonparametric test was used for the comparison of data that did not meet the requirements of the parametric test. The results of pairwise comparisons between multiple groups were corrected for significance level using the Bonferroni method. Statistical analysis and graph plotting were performed using the SPSS 26.0 software (SPSS, Chicago, IL, USA) and GraphPad Prism 8.0 (GraphPad, San Diego, CA, USA). The data of the intestinal microbiota were expressed as M (QR) or mean ± SD, and the data analysis were performed using BMK Cloud (http://www.biocloud.net, accessed on 1 October 2022). HemI 1.0 (http://hemi.biocuckoo.org/down.php, accessed on 1 November 2022) software was used to create the heatmap [23]. The *p*-value < 0.05 was judged as showing a significant difference.

## 3. Results

### 3.1. Effects of Lutein on Food Intake, Body Weight and Liver Tissue

**Figure 1:**
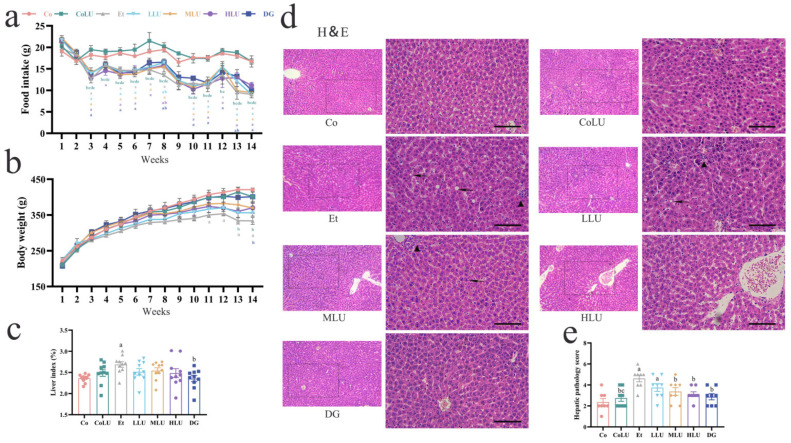
Effects of lutein on food intake, body weight and liver tissue. Changes in food intake (**a**) and body weight (**b**) during the experimental period. (**c**) Index of the liver (*n* = 10/group). Liver H&E staining (**d**) (scale bar = 50 μm, magnification 200× and 400×; black arrows indicate hepatocyte fat vacuoles, and black triangles suggest inflammatory cell aggregates; *n* = 8/group) and Hepatic pathology score (**e**) (magnification 200×; *n* = 8/group). The data were shown with mean ± SEM. Co: normal control group; CoLU: the medium-dose lutein control group; Et: ethanol model group; LLU: the low-dose lutein intervention group; MLU: the medium-dose lutein intervention group; HLU: the high-dose lutein intervention group; DG: positive control group. ^a^ *p* < 0.05 vs. Co group, ^b^ *p* < 0.05 vs. Et group, ^c^ *p* < 0.05 vs. LLU group, ^d^ *p* < 0.05 vs. MLU group, ^e^ *p* < 0.05 vs. HLU group.

At the beginning of the trial, there was no significant difference in the amount of food intake and body weight for each group. However, at week 14, the Et group’s food intake decreased more than in the Co group (*p* < 0.05, Figure 1a). In addition, the Et group had the slowest weight gain during the experiment; at week 14, the Et group had a lower weight than the Co group (*p* < 0.05). In the DG group, the rats showed an increase in body weight compared to the Et group (*p* < 0.05, Figure 1b). While the liver index showed an increase in the Et group compared with the Co group, a significant decrease in the elevated liver index caused by alcohol was observed in the DG group compared with the Et group (each *p* < 0.05, Figure 1c). The liver pathology observations showed significant pathological changes in the Et group, including disturbed hepatic cord arrangement, inflammatory cell infiltration and hepatocyte fat vacuolation. In contrast, lutein and diammonium glycyrrhizinate treatment attenuated such changes (Figure 1d). Meanwhile, the Et group showed a significant increase in liver pathology scores compared with the Co group, while MLU, HLU and DG groups reduced pathology scores compared to the Et group (each *p* < 0.05, Figure 1e).

### 3.2. Effect of Lutein on Serum Biochemical Indices

As shown in Table 3, compared with the Co group, long-term ethanol treatment increased blood levels of ALT and AST by 38.89% and 35.00%, respectively (each *p* < 0.05). In comparison with the Et group, the MLU, HLU and DG groups considerably decreased serum levels of ALT (20.90%, 22.59% and 39.21%, respectively) and AST (28.03%, 23.16% and 33.06%, respectively) (each *p* < 0.05). In comparison with the Et group, the MLU, HLU, and DG groups decreased serum levels of GGT, although the differences were not significant. Compared with the Co group, TG and TC levels in the Et group were significantly increased by 41.18% and 50.00%, respectively (each *p* < 0.05). Compared with the Et group, serum TG levels in HLU and DG groups were significantly decreased by 27.78% and 37.50%, respectively (each *p* < 0.05).

### 3.3. Effect of Lutein on Alcohol Metabolism in the Liver

**Figure 2:**
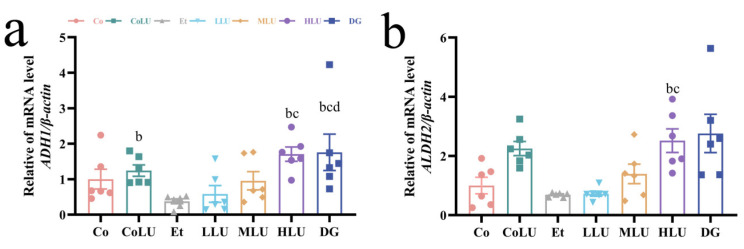
Effect of lutein on alcohol metabolism in the liver. The gene expression levels of *ADH1* (**a**) and *ALDH2* (**b**) in the liver (*n* = 6/group). The data were shown with mean ± SEM. Co: normal control group; CoLU: the medium-dose lutein control group; Et: ethanol model group; LLU: the low-dose lutein intervention group; MLU: the medium-dose lutein intervention group; HLU: the high-dose lutein intervention group; DG: positive control group. ^b^ *p* < 0.05 vs. Et group, ^c^ *p* < 0.05 vs. LLU group, ^d^ *p* < 0.05 vs. MLU group.

In this experiment, the gene expression levels of *ADH1* and *ALDH2* in liver was verified by real-time fluorescence quantitative PCR. The mRNA expression levels of *ADH1* and *ALDH2* did not differ significantly between the Co and Et groups (*p* > 0.05). However, the mRNA expression levels of *ADH1* and *ALDH2* showed a significantly greater increase in the HLU group than in the Et group (3.54-fold and 2.65-fold, respectively, each *p* < 0.05). At the same time, the mRNA expression levels of *ADH1* were increased by 2.30-fold and 3.67-fold in the CoLU and DG groups, respectively, compared with the Et group (each *p* < 0.05, Figure 2a,b).

### 3.4. Effect of Lutein on Oxidative Stress in the Liver

**Figure 3:**
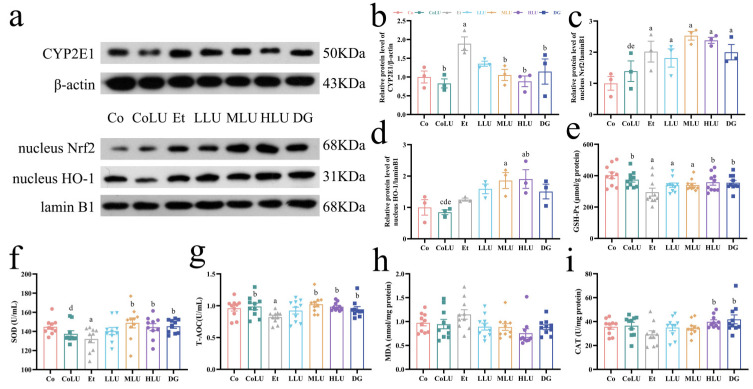
Effect of lutein on oxidative stress in the liver. (**a**) The protein expression levels of CYP2E1 (**b**), nucleus Nrf2 (**c**), and nucleus HO-1 (**d**) (*n* = 3/group). Levels of (**e**) Glutathione peroxidase (GSH-Px), (**f**) superoxide dismutase (SOD), (**g**) total antioxidant capacity (T-AOC), (**h**) malondialdehyde (MDA), (**i**) catalase (CAT) (*n* = 10/group). The data were shown with mean ± SEM. Co: normal control group; CoLU: the medium-dose lutein control group; Et: ethanol model group; LLU: the low-dose lutein intervention group; MLU: the medium-dose lutein intervention group; HLU: the high-dose lutein intervention group; DG: positive control group. ^a^ *p* < 0.05 vs. Co group, ^b^ *p* < 0.05 vs. Et group, ^c^ *p* < 0.05 vs. LLU group, ^d^ *p* < 0.05 vs. MLU group, ^e^ *p* < 0.05 vs. HLU group.

Immunoblotting was used to determine the amount of cytochrome P450 (CYP2E1) protein expression in liver tissue. Compared with the Co group, the Et group dramatically enhanced CYP2E1 protein expression by 88.80%. In comparison with the Et group, the MLU, HLU and DG groups reduced CYP2E1 expression by 44.16%, 53.15% and 39.33%, respectively (each *p* < 0.05, Figure 3a,b). The Et group upregulated the protein expression level of Nrf2 by 1.01-fold compared with the Co group (*p* < 0.05), while the expression of Nrf2 in the remaining groups, in comparison with the Et group, was not noticeably different (Figure 3c). Furthermore, compared with the Et group, the HLU group significantly increased the expression level of HO-1 by 52.49% (*p* < 0.05, Figure 3d). The levels of antioxidant factors in the organism were then examined and showed that the Et group significantly decreased the levels of GSH-Px, SOD and T-AOC in contrast to the Co group (each *p* < 0.05). Furthermore, compared to the Et group, the levels of GSH-Px significantly increased in the HLU and DG groups, and the level of SOD and T-AOC significantly increased in the MLU, HLU and DG groups (each *p* < 0.05, Figure 3e–g). In addition, lutein intervention showed a tendency to reduce MDA levels compared with the Et group, although there was no significant difference (Figure 3h). CAT levels in HLU and DG groups were significantly higher than in the Et group, as shown in Figure 3i (each *p* < 0.05).

### 3.5. Effects of Lutein on Levels of Inflammatory Cytokines

The level of inflammatory cytokines in each group was evaluated, as shown in Table 4. The Et group dramatically increased the levels of pro-inflammatory cytokines TNF-α as well as IL-1β in comparison with the Co group (26.58% and 68.26%, respectively; each *p* < 0.05). The level of TNF-α in the MLU, HLU and DG groups markedly decreased (18.99%, 15.14% and 26.42%, respectively), in comparison to the Et group, while the levels of IL-1β in the HLU and DG groups significantly decreased (23.59% and 26.24%, respectively; each *p* < 0.05). In addition, there was no significant difference in LBP levels among the groups. Serum LPS levels in the Et group were higher than in the Co group by 21.43%, while LPS levels in the HLU group were significantly lower than in the Et group by 10.64% (each *p* < 0.05).

### 3.6. Effects of Lutein on Expression of Proteins Associated with Inflammatory Pathways

**Figure 4:**
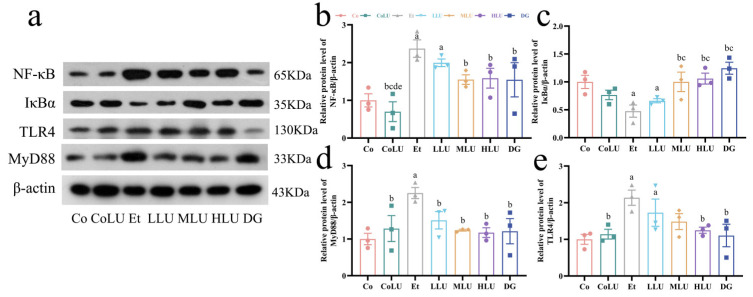
Effects of lutein on expression of proteins associated with inflammatory pathways. (**a**) The protein expression levels of NF-κB (**b**), IκB-α (**c**), MyD88 (**d**), and TLR4 (**e**) (*n* = 3/group). The data were shown with mean ± SEM. Co: normal control group; CoLU: the medium-dose lutein control group; Et: ethanol model group; LLU: the low-dose lutein intervention group; MLU: the medium-dose lutein intervention group; HLU: the high-dose lutein intervention group; DG: positive control group. ^a^ *p* < 0.05 vs. Co group, ^b^ *p* < 0.05 vs. Et group, ^c^ *p* < 0.05 vs. LLU group, ^d^ *p* < 0.05 vs. MLU group, ^e^ *p* < 0.05 vs. HLU group.

Immunoblotting revealed that the Et group drastically increased the protein expression levels of NF-κB, TLR4 as well as MyD88 in liver tissues by 1.37-fold, 1.14-fold and 1.25-fold, respectively, compared with the Co group, while decreasing the protein expression level of IκB-α by 52.39% (each *p* < 0.05). The HLU and DG groups decreased the protein expression levels of NF-κB (33.10% and 34.82%, respectively), TLR4 (41.59% and 48.36%, respectively), and MyD88 (47.81% and 46.19%, respectively) and upregulated the protein expression level of IκB-α (1.22-fold and 1.61-fold, respectively) compared with the Et group (each *p* < 0.05). Furthermore, the MLU group decreased the protein expression levels of NF-κB and MyD88 by 34.60% and 44.97%, respectively, and elevated the protein expression of IKBα by 1.10-fold, compared with the Et group, and the LLU group also reduced the protein expression level of MyD88 by 32.95% (each *p* < 0.05, Figure 4a–e).

### 3.7. Effect of Lutein on the Small Intestine Tissue

**Figure 5:**
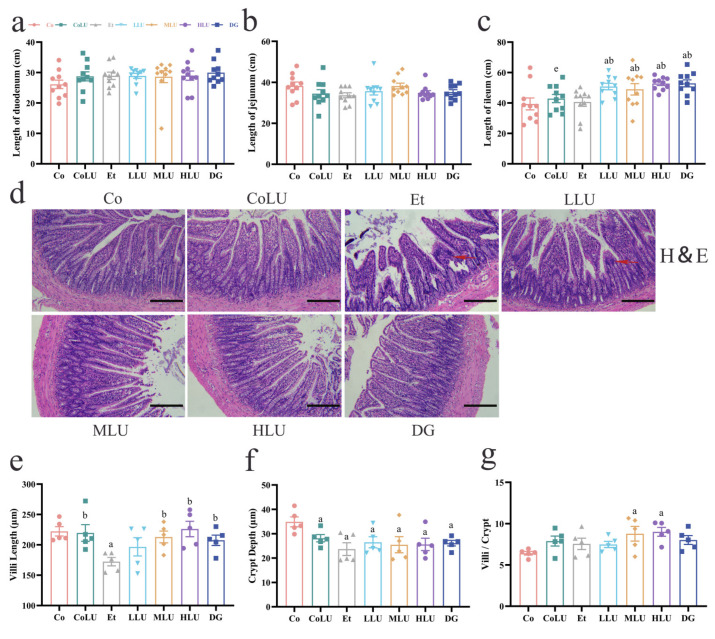
Effect of lutein on the small intestine tissue. Length of the duodenum (**a**), jejunum (**b**), and ileum (**c**) (*n* = 10/group). Ileum H&E staining (**d**) (scale bar = 50 μm, magnification 200×; red arrows indicate intestinal villi atrophy). Small intestinal villus length (**e**), crypt depth (**f**), and villus length to crypt depth ratio (**g**) (*n* = 5/group). The data were shown with mean ± SEM. Co: normal control group; CoLU: the medium-dose lutein control group; Et: ethanol model group; LLU: the low-dose lutein intervention group; MLU: the medium-dose lutein intervention group; HLU: the high-dose lutein intervention group; DG: positive control group. ^a^ *p* < 0.05 vs. Co group, ^b^ *p* < 0.05 vs. Et group, ^e^ *p* < 0.05 vs. HLU group.

Pathological changes in small intestine tissues were then analyzed. The small intestine tissue was divided into three segments: duodenum, jejunum and ileum, and the length of each segment was subsequently measured. The results showed that there was no significant difference in duodenum and jejunum lengths among all groups (Figure 5a,b), but there was a significant difference in ileum length among all groups. The ileum length of the LLU, MLU, HLU and DG groups was significantly longer than the Et group (each *p* < 0.05, Figure 5c). In addition, pathological sections of ileal tissue showed changes in ileal structure in the Et group, such as severe atrophy, rupture, and breakage of villi. After the intervention of lutein, especially high-dose administration of lutein, the pathological changes of ileal tissue were improved, and the phenomenon of intestinal villi breakage and atrophy was reduced (Figure 5d). Compared with the Co group, the villus length was significantly shortened in the Et group (*p* < 0.05, Figure 5e). The villus length was longer in the MLU, HLU and DG groups than in the Et group (each *p* < 0.05). The crypt depth was shallower in the Et group than in the Co group (*p* < 0.05, Figure 5f). The villus length to crypt depth ratio was greater in the MLU and HLU groups than in the Co group (each *p* < 0.05, Figure 5g).

### 3.8. Effect of Lutein on the Ileal Barrier

**Figure 6:**
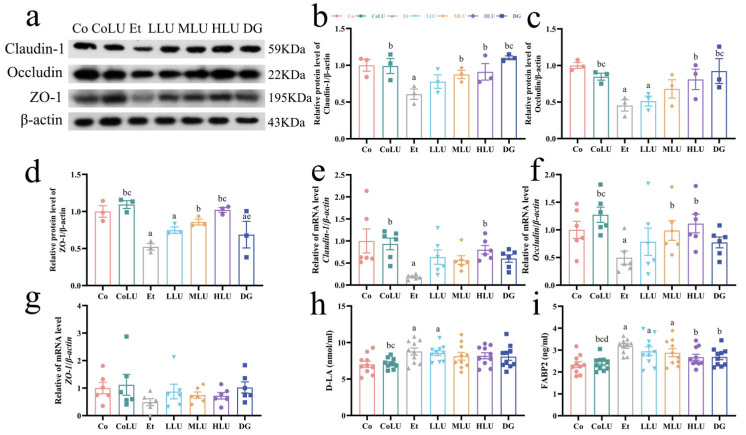
Effect of lutein on the ileal barrier. (**a**) The protein expression levels of Claudin-1 (**b**), Occludin (**c**), and ZO-1 (**d**) (*n* = 3/group). The gene expression levels of *Claudin-1* (**e**), *Occludin* (**f**), and *ZO-1* (**g**) (*n* = 6/group). Levels of (**h**) D-Lactic acid (D-LA) and (**i**) intestinal fatty acid binding protein (FABP2) (*n* = 10/group). The data were shown with mean ± SEM. Co: normal control group; CoLU: the medium-dose lutein control group; Et: ethanol model group; LLU: the low-dose lutein intervention group; MLU: the medium-dose lutein intervention group; HLU: the high-dose lutein intervention group; DG: positive control group. ^a^ *p* < 0.05 vs. Co group, ^b^ *p* < 0.05 vs. Et group, ^c^ *p* < 0.05 vs. LLU group, ^d^ *p* < 0.05 vs. MLU group, ^e^ *p* < 0.05 vs. HLU group.

This experiment verified the expression of ileal barrier proteins and genes using immunoblotting and real-time fluorescence quantitative PCR. Compared with the Co group, the protein expression levels of the intestinal barrier proteins Claudin-1, Occludin, and ZO-1 were downregulated by 39.35%, 54.70% and 47.78%, respectively, in the Et group (each *p* < 0.05). The MLU group significantly upregulated the protein expression levels of Claudin-1 as well as ZO-1 (44.24% and 64.11%, respectively), in comparison with the Et group, while the HLU group significantly upregulated the protein expression levels of Claudin-1, Occludin and ZO-1 (50.12%, 78.66% and 95.55%, respectively) (each *p* < 0.05). The protein expression levels of Claudin-1, as well as Occludin, were considerably greater in the DG group than in the Et group (0.82-fold and 1.04-fold, respectively) (each *p* < 0.05, Figure 6a–d). The mRNA expression of *Occludin* and *Claudin*-*1* was significantly decreased in the Et group compared with the Co group (50.17% and 81.61%, respectively). The mRNA expression of *Occludin* in the MLU group increased by 98.58%, and the mRNA expression levels of *Claudin*-*1* and *Occludin* in the HLU group increased by 3.36-fold and 1.24-fold, respectively, compared with the Et group (each *p* < 0.05, Figure 6e,f). However, the mRNA expression levels of *ZO-1* did not differ significantly among the groups (Figure 6g). Furthermore, the level of D-LA in the Et group was significantly higher than that in the Co group (*p* < 0.05), while low-, medium- and high-dose lutein administration did not significantly reverse the elevation of the indicator (Figure 6h). Compared with the Co group, the level of FABP2 in the Et group was significantly increased, while the level of FABP2 in the HLU and DG groups was significantly decreased compared with the Et group (*p* < 0.05, Figure 6i).

### 3.9. Effect of Lutein on the Microbiota of Cecum Contents

**Figure 7:**
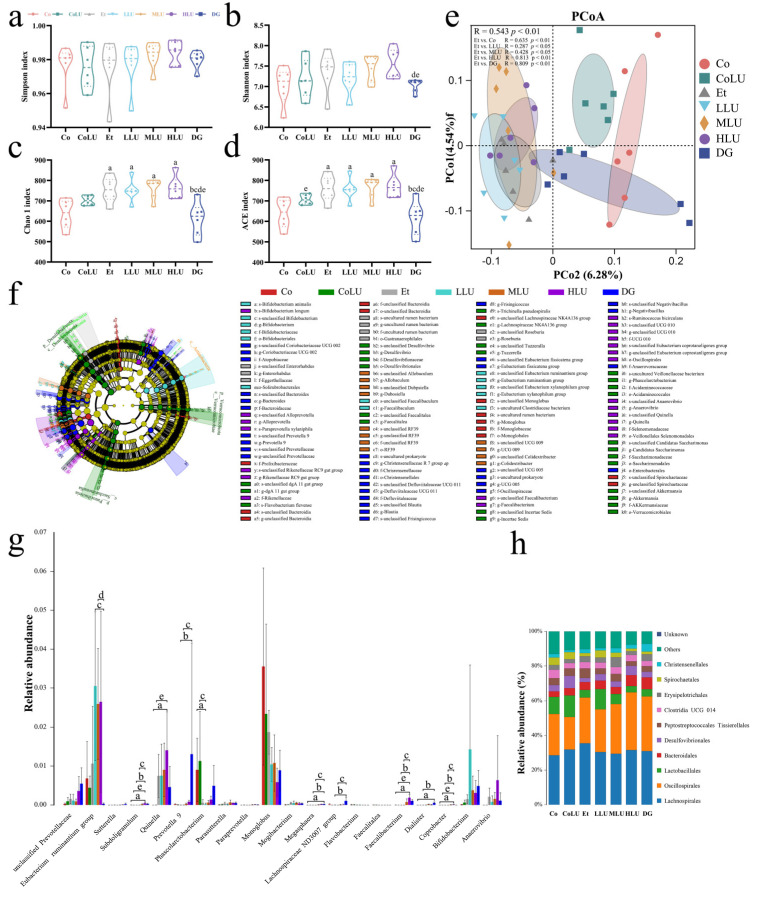
Effect of lutein on the microbiota of cecum contents. (**a**) Simpson index, (**b**) Shannon index, (**c**) Chao 1 index, (**d**) ACE index,; the data were expressed as M (QR). (**e**) PCoA based on unweighted unifrac distance (Analysis of Similarities (ANOSIM) analysis was used for comparison between groups: the closer the R-value is to 1, the more likely the difference among groups will be bigger than the difference within groups (*p* < 0.05 indicates that the test has high confidence). (**f**) LEfSe analysis (LDA score > 2.5). (**g**) The top 20 species with the most significant differences at the genus level, followed by BH-FDR correction for results with significant differences; the data were expressed as mean ± SD. (**h**) Species distribution map at the order level (*n* = 6/group). Co: normal control group; CoLU: the medium-dose lutein control group; Et: ethanol model group; LLU: the low-dose lutein intervention group; MLU: the medium-dose lutein intervention group; HLU: the high-dose lutein intervention group; DG: positive control group. ^a^ *p* < 0.05 vs. Co group, ^b^ *p* < 0.05 vs. Et group, ^c^ *p* < 0.05 vs. LLU group, ^d^ *p* < 0.05 vs. MLU group, ^e^ *p* < 0.05 vs. HLU group.

The Simpson and Shannon indexes, the measure of species diversity, and the Chao1 and ACE indexes, the measure of species abundance, were used to evaluate α-diversity among the groups. The results indicated that the Simpson index was not markedly distinguished among the groups. However, the Shannon index of MLU and HLU groups was larger than that of the DG group (*p* < 0.05, Figure 7a,b). Compared with the Co group, the Chao1 and ACE indexes in the Et group were increased (each *p* < 0.05). In contrast, the Chao1 and ACE indexes in the DG group were decreased compared with the Et group (each *p* < 0.05, Figure 7c,d). The results of PCoA analysis based on unweighted unifrac distance indicated that the microbial communities could be well separated among the groups (R = 0.543, *p* < 0.01). In addition, there was a considerable variation in β-biodiversity between the Co and Et groups (R = 0.635, *p* < 0.01), while there was also a considerable variation in β-biodiversity between the Et and each intervention group (Et vs. LLU, R = 0.287, *p* < 0.05; Et vs. MLU, R = 0.428, *p* < 0.05; Et vs. HLU, R = 0.813, *p* < 0.01; Et vs. DG, R = 0.809, *p* < 0.01; Figure 7e). Subsequently, the characteristic bacteria with significant differences in each group were analyzed using the LefSe method (LDA > 2.5, Figure 7f). In the Co group, *Monoglobus* was enriched at the genus level, and the unclassified *Lchnospiraceae* NK4A136 group was abundant at the species level (each *p* < 0.05). In the CoLU group, *Akkermansia*, *Phascolarctobacterium*, as well as *Incertae sedis,* were enriched at the genus level (each *p* < 0.05). In the Et group, the *Enterorhabdus* and uncultured *rumen bacterium* were abundant at the genus level, and *Eggerthellaceae* was enriched at the family level (each *p* < 0.05). In the LLU group, the *Eubacterium xylanophilum* group, *Faecalitales*, and *Bifidobacterium* were enriched at the genus level, and *Bifidobacterium animalis* were abundant at the species level (each *p* < 0.05). In the MLU group, *Allobaculum*, *Dubosiella*, as well as *Colidextribacter* were abundant at the genus level, while in the HLU group, *Alloprevotella*, *Faecalibacterium*, and *Quinella* were abundant at the genus level and *Bifidobacterium longum* was abundant at the species level (each *p* < 0.05). In the DG group, *Blautia*, *Coriobacteriaceae* UCG 002, *Bacteroides*, and *Negativibacillus* were enriched at the genus level (each *p* < 0.05).

Subsequently, Kruskal–Wallis tests were performed at the genus level to look for differential bacteria in each group (Figure 7g). The relative abundance of the *Eubacterium ruminantium* group was increased in the three groups following low-, medium-, and high-dose lutein intervention, with the relative abundance of this bacterium in the LLU and MLU groups being considerably higher than in the DG group (each *p* < 0.05). In the HLU group, the relative abundance of *Subdoligranulum* was considerably greater than in the Co, CoLU, Et, and LLU groups (each *p* < 0.05), the relative abundance of *Quinella* was substantially more than in the Co and CoLU groups (each *p* < 0.05), the relative abundance of *Faecalibacterium* was significantly higher than in the Co, CoLU, Et and LLU groups (each *p* < 0.05), and the relative abundance of *Coprobacter* was higher than in the Co, CoLU, Et and LLU groups (each *p* < 0.05). In the DG group, the relative abundance of *Prevotella* 9 was considerably more than in the Et and LLU groups (each *p* < 0.05), the relative abundance of *Megasphaera* was substantially more than in the Co, Et, and LLU groups (each *p* < 0.05), the relative abundance of *Lachnospiraceae* ND3007 group was substantially higher than in the Et and LLU groups (each *p* < 0.05), and the relative abundance of *Dialister* was substantially higher than in the Co, Et and LLU groups (each *p* < 0.05). The relative abundance of *Phascolarctobacterium* was substantially lower in the LLU group than in the Co and CoLU groups (each *p* < 0.05). The species distribution of cecum contents at the order level for each group is shown in Figure 7h.

### 3.10. Correlation of Differential Bacterial Genera with Biochemical Indicators

**Figure 8:**
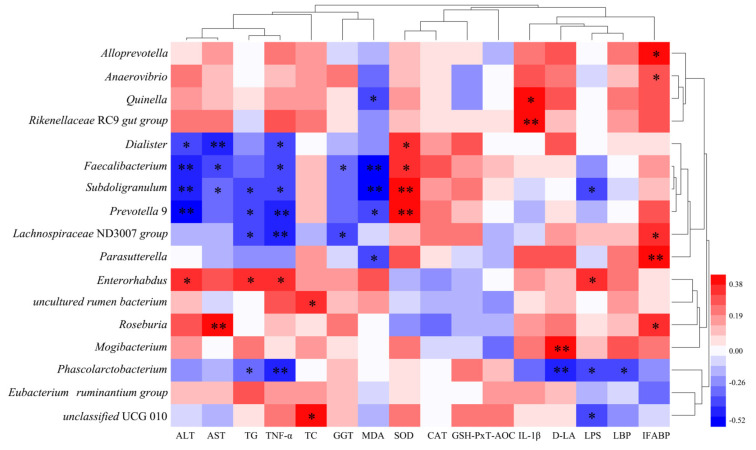
Correlation of differential bacterial genera with biochemical indicators. Differential bacterial genera were defined as genera with relative abundance above 0.01% in each group, including differential genera identified using Kruskal-Wallis tests for each group with BH-FDR correction for significance results. Genera identified by LEfSe analysis (LDA > 2.5) were significantly enriched in the Et and HLU groups, respectively (*p* < 0.05) (*n* = 6/group). (Spearman correlation analysis: correlation coefficient threshold set to 0.1, positive correlation in red, negative correlation in blue). * *p* < 0.05, ** *p* < 0.01.

As shown in Figure 8, *Enterorhabdus* was positively correlated with ALT (*r* = 0.35, *p* < 0.05), TNF-α (*r* = 0.37, *p* < 0.05), TG (*r* = 0.36, *p* < 0.05), and LPS (*r* = 0.32, *p* < 0.05). *Faecalibacterium* was negatively correlated with ALT (*r* = −0.43, *p* < 0.01), AST (*r* = −0.37, *p* < 0.05), TNF-α (*r* = −0.35, *p* < 0.05), GGT (*r* = −0.32, *p* < 0.01), and MDA (*r* = −0.52, *p* < 0.05) and positively correlated with SOD (*r* = 0.35, *p* < 0.05). *Subdoligranulum* was negatively correlated with ALT (*r* = 0.41, *p* < 0.01), AST (*r* = −0.33, *p* < 0.05), TG (*r* = −0.35, *p* < 0.05), TNF-α (*r* = −0.38, *p* < 0.05), LPS (*r* = −0.36, *p* < 0.05), and MDA (*r* = −0.52, *p* < 0.01) and positively correlated with SOD (*r* = 0.40, *p* < 0.01). *Phascolarctobacterium* was negatively correlated with TNF-α (*r* = −0.40, *p* < 0.01), TG (*r* = −0.31, *p* < 0.05), LPS (*r* = −0.36, *p* < 0.05), LBP (*r* = −0.36, *p* < 0.05), and D-LA (*r* = −0.41, *p* < 0.01).

## 4. Discussion

The current study investigated the role and mechanism of lutein in the liver and intestinal barrier damage in rats induced by persistent alcohol. The findings revealed that lutein might reduce liver and intestinal barrier damage in rats via antioxidant and anti-inflammatory actions and enrich beneficial flora in rats.

Chronic alcohol consumption may lead to disturbances in the nutritional metabolism of the organism [24], causing slow weight gain in experimental rats [25]. Consistent with previous studies, our alcohol-fed rats showed a phenomenon of reduced food intake and weight, possibly because alcohol intake causes anorexia in rats [26], which increases the risk of malnutrition, thus causing low body weight. Meanwhile, the levels of transaminases (ALT, AST) in serum were raised as a result of hepatocyte damage which was induced by ethanol [27,28], whereas high-dose lutein intervention attenuated alcohol-induced transaminase elevation, demonstrating the protective effect of lutein on hepatocytes [15,29]. In addition, long-term alcohol intake can promote TG and TC synthesis and hepatocyte steatosis in the organism [30,31], leading to increased serum TG and TC levels and a corresponding increase in the liver index [32]. Lutein can prevent excessive lipid accumulation [33] and also effectively ameliorate liver damage in rats with nonalcoholic fatty liver [12]. Consistent with this, supplementation with high-dose lutein significantly reduced TG levels in chronic-alcohol-consuming rats in the current work.

Liver is the main site of ethanol metabolism, and alcohol dehydrogenase (ADH), aldehyde dehydrogenase 2 (ALDH2), and cytochrome P450 2E1 (CYP2E1) in liver cells are closely related to alcohol metabolism [34]. Long-term alcohol intake can reduce the activities of ADH1 and ALDH2 and increase CYP2E1 protein expression in the liver, and this generates excessive reactive oxygen species (ROS) that induce liver damage [4,35,36]. In particular, the massive accumulation of ROS disrupts lipid metabolism in the liver and elevates MDA, the end product of lipid peroxidation, as well as TG levels [37,38]. After high-dose lutein intervention, the mRNA expression of *ALDH2* related to alcohol metabolism in the liver was promoted, which may be related to the fact that antioxidants can promote alcohol metabolism and resist alcohol-induced liver injury by regulating the feedback mechanism between Nrf2 and ALDH2 [39]. Despite this, there was no significant difference in Nrf2 expression between the Et group and the HLU group in this study. However, high-dose lutein intervention promoted the expression of HO-1, an antioxidative factor downstream of Nrf2, so lutein may promote alcohol metabolism by playing an antioxidative role, but further studies may be needed to explore the relationship between lutein and alcohol metabolism. Chronic alcohol feeding may elevate the protein levels of Nrf2 in the liver cell nuclei of experimental rats, which may be a compensatory response [40,41]. However, long-term alcohol consumption did not increase the expression of the antioxidative factor HO-1, thereby also reducing the generation of the enzymatic antioxidative factors GSH-Px and SOD, which resist the excessive accumulation of ROS in the organism [42,43,44]. Meanwhile, chronic alcohol intake depletes the body of enzymatic antioxidative factors [45], thus creating a vicious circle and promoting disease progression. However, the lutein intervention reduced the alcohol-induced elevated protein expression level of CYP2E1, while further alleviating the levels of TG and MDA, suggesting that lutein may have ameliorated liver injury through antioxidative effects. At the same time, administration of high-dose lutein further promoted the expression of the antioxidative factor HO-1, thus resisting the alcohol-induced decrease in the levels of antioxidative factors SOD and GSH-Px, and also has the potential to raise T-AOC, which can reflect the organism’s non-enzymatic antioxidative capacity [46]. In conclusion, lutein may play an antioxidative role in antagonizing alcohol-induced liver injury by regulating the Nrf2/HO-1 signaling pathway.

Chronic alcohol intake causes inflammatory damage and oxidative stress in the liver. Ethanol and its metabolites can induce dissociation of the IKK complex (composed of NF-κB and IκB-α) and degrade IκB-α, thereby dissociating the complex to release NF-κB as well as promoting the release of inflammatory factors, such as TNF-α and IL-1β, further aggravating liver damage [47]. Furthermore, chronic alcohol consumption leads to elevated blood levels of the endotoxin LPS, which is one of the central mediators of alcoholic hepatitis [48]. The lipopolysaccharide-binding protein (LBP) in the blood binds to intestinal-derived LPS, which can transport it to the liver and induce inflammatory damage in the liver [49]. Specifically, upon entering the liver, LPS first binds to the recognition receptor Toll-like receptor 4 (TLR4) [50], which, when the TLR4-MyD88 complex is formed, can also subsequently release NF-κB as well as increase the levels of its downstream inflammatory factors [51,52], further aggravating the liver injury. In the present study, chronic ethanol intake activated the inflammatory pathway in the liver and thus promoted the release of the pro-inflammatory factors TNF-α as well as IL-1β, leading to inflammatory damage in the liver. This liver damage may be caused by the combined action of alcohol through its metabolites and the LPS pathway. This study also found that supplementation with lutein reversed alcohol-induced changes in inflammatory protein expression, reduced the level of inflammatory factors, and ameliorated liver injury to some extent. Oxidative stress and inflammatory responses are closely related pathological alterations, and there is mutual crosstalk between Nrf2 and NF-κB [53], so lutein may be acting in joint resistance to alcohol-induced liver damage by exerting antioxidative and anti-inflammatory capacities.

Meanwhile, chronic alcohol intake can induce oxidative stress in the intestine [54], causing increased intestinal permeability and disrupting the intestinal tight junction [5], which consists of transmembrane proteins such as Occludins and Claudins as well as intracellular molecules such as ZO-1 [55]. At the same time, intestinal tight junction disruption leads to intestinal barrier breakdown, which promotes the release of LPS and further causes liver damage [48]. Previous studies have found that intestinal leakage after chronic alcohol exposure occurs in the ileum, but not in the duodenum or jejunum [56]. Therefore, we examined the tight junctions of the ileum. We found that chronic alcohol intake disrupts the tight junctions of the ileum, leading to decreased protein levels of Claudin-1, Occludin and ZO-1, and considerably elevating the level of I-FABP, which is positively correlated with intestinal permeability [57], resulting in an impaired intestinal barrier. Previous studies had found that lutein can reduce small intestinal villus injury and intestinal cell shedding, inhibit the increase of lipid peroxide, and improve intestinal oxidative injury caused by ischemia-reperfusion (I/R) [58,59]. In addition, lutein could improve intestinal oxidative stress damage by exerting antioxidative effects [60], and it can also regulate intestinal tight junction opening and improve the impaired gut barrier [16]. In this research, supplementation with high-dose lutein up-regulated the protein and gene expression of Claudin-1 and Occludin and alleviated the high FABP2 status in the organism, which improved the impaired intestinal barrier. By supplementing with lutein, the LPS level was reduced, and the liver inflammation caused by alcohol was further alleviated.

Furthermore, alcohol intake can lead to bacterial overgrowth in the cecum [61]. The current study found that the Chao1 and ACE indexes, which measure the number of bacteria, were increased after alcohol intake, indicating that alcohol caused bacterial overgrowth in the cecum. The Et group was significantly enriched for *Eggerthellaceae*, a pathogenic bacterium [62], and *Enterorhabdus*, a Gram-negative bacterium, in which LPS is a part of the cell wall of Gram-negative bacteria. Since alcohol causes a leaky gut, it further promotes the increase of LPS in the blood and aggravates liver damage. In addition, *Enterorhabdus* were positively correlated with factors that could promote the release of pro-inflammatory cytokines and damage the epithelial barrier [63]. In contrast, after high-dose lutein intervention, the intestinal flora composition changed dramatically (Et vs. HLU, R = 0.813, *p* < 0.01), although the Chao1 and ACE index did not significantly alter. In contrast to the Et group, the HLU group was enriched for *Bifidobacterium longum*, which may create short-chain fatty acids [64]. In addition, *Bifidobacterium longum* has been found to improve alcohol-induced liver and intestinal barrier damage [65]; this also implies that, by enriching *Bifidobacterium longum*, lutein intervention might reduce alcohol-induced damage in the body. Previous research has discovered that prolonged excessive alcohol use reduces the relative abundance of *Subdoligranulum* and *Faecalibacterium* [66,67], particularly when intestinal permeability is high [68]. In the current research, however, the administration of high-dose lutein intervention improved alcohol-induced intestinal barrier damage and reduced intestinal permeability, while the relative abundance of *Subdoligranulum*, as well as *Faecalibacterium*, was increased, which also further indicated the regulatory effect of lutein on alcohol-induced intestinal barrier injury and flora disorders.

A previous study has found that lutein can improve liver injury induced by binge drinking in rats by inducing antioxidative and anti-inflammatory effects [69]. The present study investigated the protective effect of lutein on liver injury induced by chronic alcohol intake. Our study focused on the dose-response relationship and the combined effects of the liver–gut axis, and also explored the relationship between lutein and gut microbiota, which were not examined in the previous study. The mechanisms of liver injury induced by acute and chronic alcohol feeding were similar but different. Changes in gene expression after long-term ethanol feeding might sensitize the liver to alcohol-induced damage that is not seen after acute alcoholism [70]. At the same time, long-term alcohol feeding can lead to increased protein expression of CYP2E1, and intestinal permeability will also increase with the long-term consumption of ethanol, at which time TLR4-mediated liver inflammatory injury is particularly important [71,72,73]. The previous study has found that short-term high alcohol intake induces oxidative stress and inflammatory responses in the liver. Lutein intervention enhanced the protein expression of Nrf2 in the liver of rats, up-regulated the level of antioxidants in the body, down-regulated the protein expression of NF-κB and its downstream target factors COX-2 and iNOS, and reduced the level of inflammation-related factors [69]. However, in the present study, lutein administration did not significantly increase the expression of Nrf2 in the liver, which may be related to the continuous oxidative stress in the body caused by long-term alcohol exposure. However, lutein administration could promote the protein expression of the antioxidative factor HO-1 downstream of Nrf2, regulate the NF-κB/TLR4 inflammatory pathway, and alleviate the damage to the intestinal barrier, thereby reducing the oxidative and inflammatory damage to the liver and ileum caused by long-term alcohol intake. Recent studies have found that autophagy plays an important role in the pathology of alcoholic liver disease, as acute alcohol intake enhances autophagy in the liver and, conversely, chronic alcohol feeding inhibits autophagy, although many conflicting studies have emerged [74,75,76]. However, existing studies have found that lutein can induce protective autophagy in cells under stress, while at the same time having a resistance effect on harmful excessive autophagy in cells [77,78]. Although the relationship between lutein and autophagy in the liver under continuous alcohol intake was not investigated in this study, it has provided ideas for future research.

## 5. Conclusions

In conclusion, in the current trial, liver injury and intestinal barrier dysfunction induced by chronic alcohol use in rats were attenuated by lutein. Moreover, the protective effect of lutein is connected to its regulation of the Nrf2/HO-1 pathway and the TLR4/NF-κB pathway. Furthermore, high-dose lutein supplementation has the potential to enrich beneficial flora (e.g., *Bifidobacterium longum*, *Faecalibacterium*, and *Subdoligranulum*). This animal study implies that lutein might be utilized as a dietary supplement to prevent and alleviate chronic alcoholic liver damage and intestinal barrier dysfunction; however, further human studies are needed to validate this.

## Figures and Tables

**Table 1:** The pathological score of the liver.

Scheme	Scoring Criteria	
	The Percentage of Cells that Contain Fat	The Number of Lesions with Inflammation or Cell Necrosis
1	≤25%	1
2	26 to 50%	≥2
3	51 to 75%	
4	≥75%	

**Table 2:** The sequences of the gene primers.

Primer Name	Sequence
Liver-*ADH1*	Forward (5′–3′) GGTGTTGGTCTGTCTGTCGT Reverse (5′–3′) ATGGTGTCAAGACGGCCAAT
Liver-*ALDH2*	Forward (5′–3′) GCTGACAAGTACCACGGGAA Reverse (5′–3′) CACGTTTCCAGTTGCCAAGG
Ileum-*Claudin-1*	Forward (5′–3′) GGCTTCGCTGGGATGGATCG Reverse (5′–3′) TGCACTGTATCTGCCCGGTG
Ileum-*Occludin*	Forward (5′–3′) TGTGCTCACAGGTGGTTGCC Reverse (5′–3′) AGACCAAACTGGGCTGGATGC
Ileum-*ZO-1*	Forward (5′–3′) ACAAGCGCAGCCACAAGCTA Reverse (5′–3′) TGGGCTCCTCCAGGTTGACA
*β-actin*	Forward (5′–3′) AAGTGCGACGTGGACATCCG Reverse (5′–3′) GGGCGGTGATCTCCTTCTGC

**Table 3:** Effect of Lutein on Serum Biochemical Indices.

Group	ALT (U/L)	AST (U/L)	GGT (U/L)	TG (mmol/L)	TC (mmol/L)
Co	46.24 ± 2.35	38.34 ± 1.50	1.90 ± 0.28	0.51 ± 0.04	1.52 ± 0.07
CoLU	50.10 ± 4.33 ^b^	35.34 ± 3.17 ^bc^	2.10 ± 0.55	0.61 ± 0.07	1.70 ± 0.17
Et	64.22 ± 5.87 ^a^	51.76 ± 2.68 ^a^	3.10 ± 0.62	0.72 ± 0.07 ^a^	2.28 ± 0.25 ^a^
LLU	58.02 ± 5.33	45.26 ± 2.19	2.50 ± 0.64	0.62 ± 0.04	1.54 ± 0.09
MLU	50.80 ± 5.63 ^b^	37.25 ± 3.41 ^bc^	1.30 ± 0.30	0.62 ± 0.06	1.51 ± 0.12
HLU	49.71 ± 5.41 ^b^	39.77 ± 3.15 ^b^	1.50 ± 0.31	0.52 ± 0.05 ^b^	1.87 ± 0.17
DG	39.04 ± 2.79 ^bc^	34.65±1.40 ^bc^	1.30 ± 0.21	0.45 ± 0.03 ^bcd^	1.69 ± 0.11

Note: ALT: Alanine aminotransferase; AST: Aspartate aminotransferase; GGT: Gamma-glutamyl transferase; TG: Triglycerides; TC: Total cholesterol. The data were shown with mean ± SEM, *n* = 10/group. Co: normal control group; CoLU: the medium-dose lutein control group; Et: ethanol model group; LLU: the low-dose lutein intervention group; MLU: the medium-dose lutein intervention group; HLU: the high-dose lutein intervention group; DG: positive control group. ^a^ *p* < 0.05 vs. Co group, ^b^ *p* < 0.05 vs. Et group, ^c^ *p* < 0.05 vs. LLU group, ^d^ *p* < 0.05 vs. MLU group.

**Table 4:** Effects of lutein on levels of inflammatory cytokines.

Group	TNF-α (pg/mL)	IL-1β (pg/mL)	LBP (mg/mL)	LPS (ng/mL)
Co	9.03 ± 0.54	18.87 ± 1.14	1.38 ± 0.13	143.57 ± 8.27
CoLU	9.52 ± 0.33 ^b^	24.49 ± 2.60 ^b^	1.41 ± 0.08	158.05 ± 6.86
Et	11.43 ± 1.08 ^a^	31.75 ± 3.26 ^a^	1.85 ± 0.09	174.33 ± 6.81 ^a^
LLU	10.25 ± 0.52	27.01 ± 2.92 ^a^	1.67 ± 0.16	166.31 ± 7.14 ^a^
MLU	9.26 ± 0.50 ^b^	27.78 ± 2.69 ^a^	1.60 ± 0.12	158.79 ± 5.70
HLU	9.70 ± 0.54 ^b^	24.26 ± 1.58 ^b^	1.48 ± 0.12	155.79 ± 3.87 ^b^
DG	8.41 ± 0.42 ^bc^	23.42 ± 2.99 ^b^	1.47 ± 0.16	165.63 ± 5.54 ^a^

Note: TNF-α: Tumor necrosis factor α; IL-1β: Interleukin 1β; LBP: Lipopolysaccharide binding protein; LPS: Lipopolysaccharide. The data were shown with mean ± SEM, *n* = 10/group. Co: normal control group; CoLU: the medium-dose lutein control group; Et: ethanol model group; LLU: the low-dose lutein intervention group; MLU: the medium-dose lutein intervention group; HLU: the high-dose lutein intervention group; DG: positive control group. ^a^ *p* < 0.05 vs. Co group, ^b^ *p* < 0.05 vs. Et group, ^c^ *p* < 0.05 vs. LLU group.

## Data Availability

The paper and Supplementary Materials contain database names and accession numbers. This study’s datasets are now available in internet databases. The NCBI database login number is PRJNA879916.

## Supplementary Materials

The following supporting information can be downloaded at: https://www.mdpi.com/article/10.3390/nu15051229/s1. See the “Supplementary Material” for detailed procedures for the extraction of cytoplasmic or nuclear proteins from liver and or ileal tissues.

## References

[1] Seitz H.K., Bataller R., Cortez-Pinto H., Gao B., Gual A., Lackner C., Mathurin P., Mueller S., Szabo G., Tsukamoto H. (2018). Alcoholic liver disease. Nat. Rev. Dis. Prim..

[2] Rocco A., Compare D., Angrisani D., Sanduzzi Zamparelli M., Nardone G. (2014). Alcoholic disease: Liver and beyond. World J. Gastroenterol..

[3] Lu Y., Zhuge J., Wang X., Bai J., Cederbaum A.I. (2008). Cytochrome P450 2E1 contributes to ethanol-induced fatty liver in mice. Hepatology.

[4] Lu Y., Cederbaum A.I. (2008). CYP2E1 and oxidative liver injury by alcohol. Free Radic. Biol. Med..

[5] Forsyth C.B., Farhadi A., Jakate S.M., Tang Y., Shaikh M., Keshavarzian A. (2009). Lactobacillus GG treatment ameliorates alcohol-induced intestinal oxidative stress, gut leakiness, and liver injury in a rat model of alcoholic steatohepatitis. Alcohol.

[6] Malaguarnera G., Giordano M., Nunnari G., Bertino G., Malaguarnera M. (2014). Gut microbiota in alcoholic liver disease: Pathogenetic role and therapeutic perspectives. World J. Gastroenterol..

[7] Ghorbani Z., Hajizadeh M., Hekmatdoost A. (2016). Dietary supplementation in patients with alcoholic liver disease: A review on current evidence. Hepatobiliary Pancreat. Dis. Int..

[8] Ahn Y.J., Kim H. (2021). Lutein as a Modulator of Oxidative Stress-Mediated Inflammatory Diseases. Antioxidants.

[9] Chung H.Y., Rasmussen H.M., Johnson E.J. (2004). Lutein bioavailability is higher from lutein-enriched eggs than from supplements and spinach in men. J. Nutr..

[10] Alves-Rodrigues A., Shao A. (2004). The science behind lutein. Toxicol. Lett..

[11] Ranard K.M., Jeon S., Mohn E.S., Griffiths J.C., Johnson E.J., Erdman J.W. (2017). Dietary guidance for lutein: Consideration for intake recommendations is scientifically supported. Eur. J. Nutr..

[12] Qiu X., Gao D.H., Xiang X., Xiong Y.F., Zhu T.S., Liu L.G., Sun X.F., Hao L.P. (2015). Ameliorative effects of lutein on non-alcoholic fatty liver disease in rats. World J. Gastroenterol..

[13] Li S., Ding Y., Niu Q., Xu S., Pang L., Ma R., Jing M., Feng G., Tang J.X., Zhang Q. (2015). Lutein has a protective effect on hepatotoxicity induced by arsenic via Nrf2 signaling. BioMed Res. Int..

[14] Kim J.H., Na H.J., Kim C.K., Kim J.Y., Ha K.S., Lee H., Chung H.T., Kwon H.J., Kwon Y.G., Kim Y.M. (2008). The non-provitamin A carotenoid, lutein, inhibits NF-kappaB-dependent gene expression through redox-based regulation of the phosphatidylinositol 3-kinase/PTEN/Akt and NF-kappaB-inducing kinase pathways: Role of H_2_O_2_ in NF-kappaB activation. Free Radic. Biol. Med..

[15] Sindhu E.R., Firdous A.P., Preethi K.C., Kuttan R. (2010). Carotenoid lutein protects rats from paracetamol-, carbon tetrachloride- and ethanol-induced hepatic damage. J. Pharm. Pharmacol..

[16] Nagira M., Tomita M., Mizuno S., Kumata M., Ayabe T., Hayashi M. (2006). Ischemia/reperfusion injury in the monolayers of human intestinal epithelial cell line caco-2 and its recovery by antioxidants. Drug Metab. Pharmacokinet..

[17] Vieira M.M., Paik J., Blaner W.S., Soares A.M., Mota R.M., Guerrant R.L., Lima A.A. (2008). Carotenoids, retinol, and intestinal barrier function in children from northeastern Brazil. J. Pediatr. Gastroenterol. Nutr..

[18] Gao M., Li X., He L., Yang J., Ye X., Xiao F., Wei H. (2019). Diammonium Glycyrrhizinate Mitigates Liver Injury Via Inhibiting Proliferation of NKT Cells And Promoting Proliferation of Tregs. Drug Des. Dev. Ther..

[19] Ouyang B., Li Z., Ji X., Huang J., Zhang H., Jiang C. (2019). The protective role of lutein on isoproterenol-induced cardiac failure rat model through improving cardiac morphology, antioxidant status via positively regulating Nrf2/HO-1 signalling pathway. Pharm. Biol..

[20] Ge N., Liang H., Zhao Y.Y., Liu Y., Gong A.J., Zhang W.L. (2018). Aplysin Protects Against Alcohol-Induced Liver Injury Via Alleviating Oxidative Damage and Modulating Endogenous Apoptosis-Related Genes Expression in Rats. J. Food Sci..

[21] Lu M., Sun J., Zhao Y., Zhang H., Li X., Zhou J., Dang H., Zhang J., Huang W., Qi C. (2022). Prevention of High-Fat Diet-Induced Hypercholesterolemia by Lactobacillus reuteri Fn041 Through Promoting Cholesterol and Bile Salt Excretion and Intestinal Mucosal Barrier Functions. Front. Nutr..

[22] Cui M., Qi C., Yang L., Zhang M., Wang H., She G., Yu R., Miao T., Sun J. (2020). A pregnancy complication-dependent change in SIgA-targeted microbiota during third trimester. Food Funct..

[23] Deng W., Wang Y., Liu Z., Cheng H., Xue Y. (2014). HemI: A toolkit for illustrating heatmaps. PLoS ONE.

[24] Lieber C.S. (2003). Relationships between nutrition, alcohol use, and liver disease. Alcohol Res. Health.

[25] Aruna K., Rukkumani R., Varma P.S., Menon V.P. (2005). Therapeutic role of Cuminum cyminum on ethanol and thermally oxidized sunflower oil induced toxicity. Phytother. Res. PTR.

[26] Kołota A., Głąbska D., Oczkowski M., Gromadzka-Ostrowska J. (2019). Influence of Alcohol Consumption on Body Mass Gain and Liver Antioxidant Defense in Adolescent Growing Male Rats. Int. J. Environ. Res. Public Health.

[27] Cao Y.W., Jiang Y., Zhang D.Y., Wang M., Chen W.S., Su H., Wang Y.T., Wan J.B. (2015). Protective effects of Penthorum chinense Pursh against chronic ethanol-induced liver injury in mice. J. Ethnopharmacol..

[28] Whitfield J.B. (2001). Gamma glutamyl transferase. Crit. Rev. Clin. Lab. Sci..

[29] El-Kholy A.A., Elkablawy M.A., El-Agamy D.S. (2017). Lutein mitigates cyclophosphamide induced lung and liver injury via NF-κB/MAPK dependent mechanism. Biomed. Pharmacother..

[30] You M., Arteel G.E. (2019). Effect of ethanol on lipid metabolism. J. Hepatol..

[31] Wang Z., Su B., Fan S., Fei H., Zhao W. (2015). Protective effect of oligomeric proanthocyanidins against alcohol-induced liver steatosis and injury in mice. Biochem. Biophys. Res. Commun..

[32] Zhao H., Liu S., Zhao H., Liu Y., Xue M., Zhang H., Qiu X., Sun Z., Liang H. (2021). Protective effects of fucoidan against ethanol-induced liver injury through maintaining mitochondrial function and mitophagy balance in rats. Food Funct..

[33] Wang N., Wang D., Luo G., Zhou J., Tan Z., Du Y., Xie H., Liu L., Yang X., Hao L. (2021). Lutein attenuates excessive lipid accumulation in differentiated 3T3-L1 cells and abdominal adipose tissue of rats by the SIRT1-mediated pathway. Int. J. Biochem. Cell Biol..

[34] Cioarca-Nedelcu R., Atanasiu V., Stoian I. (2021). Alcoholic liver disease-from steatosis to cirrhosis—A biochemistry approach. J. Med. Life.

[35] Butura A., Nilsson K., Morgan K., Morgan T.R., French S.W., Johansson I., Schuppe-Koistinen I., Ingelman-Sundberg M. (2009). The impact of CYP2E1 on the development of alcoholic liver disease as studied in a transgenic mouse model. J. Hepatol..

[36] Chen S., Huang Y., Su H., Zhu W., Wei Y., Long Y., Shi Y., Wei J. (2022). The Integrated Analysis of Transcriptomics and Metabolomics Unveils the Therapeutical Effect of Asiatic Acid on Alcoholic Hepatitis in Rats. Inflammation.

[37] Wang S., Wan T., Ye M., Qiu Y., Pei L., Jiang R., Pang N., Huang Y., Liang B., Ling W. (2018). Nicotinamide riboside attenuates alcohol induced liver injuries via activation of SirT1/PGC-1α/mitochondrial biosynthesis pathway. Redox Biol..

[38] Yin H., Hu M., Liang X., Ajmo J.M., Li X., Bataller R., Odena G., Stevens S.M., You M. (2014). Deletion of SIRT1 from hepatocytes in mice disrupts lipin-1 signaling and aggravates alcoholic fatty liver. Gastroenterology.

[39] Zhang H., Xue L., Li B., Zhang Z., Tao S. (2019). Vitamin D Protects Against Alcohol-Induced Liver Cell Injury within an NRF2-ALDH2 Feedback Loop. Mol. Nutr. Food Res..

[40] Rejitha S., Prathibha P., Indira M. (2015). Nrf2-mediated antioxidant response by ethanolic extract of Sida cordifolia provides protection against alcohol-induced oxidative stress in liver by upregulation of glutathione metabolism. Redox Rep. Commun. Free Radic. Res..

[41] Gong P., Cederbaum A.I. (2006). Nrf2 is increased by CYP2E1 in rodent liver and HepG2 cells and protects against oxidative stress caused by CYP2E1. Hepatology.

[42] Cohen J.I., Roychowdhury S., DiBello P.M., Jacobsen D.W., Nagy L.E. (2009). Exogenous thioredoxin prevents ethanol-induced oxidative damage and apoptosis in mouse liver. Hepatology.

[43] Hansen J.M., Watson W.H., Jones D.P. (2004). Compartmentation of Nrf-2 redox control: Regulation of cytoplasmic activation by glutathione and DNA binding by thioredoxin-1. Toxicol. Sci..

[44] Mandal P., Park P.H., McMullen M.R., Pratt B.T., Nagy L.E. (2010). The anti-inflammatory effects of adiponectin are mediated via a heme oxygenase-1-dependent pathway in rat Kupffer cells. Hepatology.

[45] Das S.K., Vasudevan D.M. (2007). Alcohol-induced oxidative stress. Life Sci..

[46] Wang X., Liu M., Zhang C., Li S., Yang Q., Zhang J., Gong Z., Han J., Jia L. (2018). Antioxidant Activity and Protective Effects of Enzyme-Extracted *Oudemansiella radiata* Polysaccharides on Alcohol-Induced Liver Injury. Molecules.

[47] Wang B., Gao X., Liu B., Li Y., Bai M., Zhang Z., Xu E., Xiong Z., Hu Y. (2019). Protective effects of curcumin against chronic alcohol-induced liver injury in mice through modulating mitochondrial dysfunction and inhibiting endoplasmic reticulum stress. Food Nutr. Res..

[48] Szabo G. (2015). Gut-liver axis in alcoholic liver disease. Gastroenterology.

[49] Uesugi T., Froh M., Arteel G.E., Bradford B.U., Wheeler M.D., Gäbele E., Isayama F., Thurman R.G. (2002). Role of lipopolysaccharide-binding protein in early alcohol-induced liver injury in mice. J. Immunol..

[50] Boutagy N.E., McMillan R.P., Frisard M.I., Hulver M.W. (2016). Metabolic endotoxemia with obesity: Is it real and is it relevant?. Biochimie.

[51] Nowak A.J., Relja B. (2020). The Impact of Acute or Chronic Alcohol Intake on the NF-κB Signaling Pathway in Alcohol-Related Liver Disease. Int. J. Mol. Sci..

[52] Takeda K., Akira S. (2004). TLR signaling pathways. Semin. Immunol..

[53] Wardyn J.D., Ponsford A.H., Sanderson C.M. (2015). Dissecting molecular cross-talk between Nrf2 and NF-κB response pathways. Biochem. Soc. Trans..

[54] Keshavarzian A., Farhadi A., Forsyth C.B., Rangan J., Jakate S., Shaikh M., Banan A., Fields J.Z. (2009). Evidence that chronic alcohol exposure promotes intestinal oxidative stress, intestinal hyperpermeability and endotoxemia prior to development of alcoholic steatohepatitis in rats. J. Hepatol..

[55] Förster C. (2008). Tight junctions and the modulation of barrier function in disease. Histochem. Cell Biol..

[56] Zhong W., McClain C.J., Cave M., Kang Y.J., Zhou Z. (2010). The role of zinc deficiency in alcohol-induced intestinal barrier dysfunction. Am. J. Physiol. Gastrointest. Liver Physiol..

[57] March D.S., Marchbank T., Playford R.J., Jones A.W., Thatcher R., Davison G. (2017). Intestinal fatty acid-binding protein and gut permeability responses to exercise. Eur. J. Appl. Physiol..

[58] Sato Y., Kobayashi M., Itagaki S., Hirano T., Noda T., Mizuno S., Sugawara M., Iseki K. (2011). Protective effect of lutein after ischemia-reperfusion in the small intestine. Food Chem..

[59] Ogura W., Itagaki S., Kurokawa T., Noda T., Hirano T., Mizuno S., Iseki K. (2006). Protective effect of lutein on ischemia-reperfusion injury in rat small intestine. Biol. Pharm. Bull..

[60] Chang C.J., Lin J.F., Chang H.H., Lee G.A., Hung C.F. (2013). Lutein protects against methotrexate-induced and reactive oxygen species-mediated apoptotic cell injury of IEC-6 cells. PLoS ONE.

[61] Yan A.W., Fouts D.E., Brandl J., Stärkel P., Torralba M., Schott E., Tsukamoto H., Nelson K.E., Brenner D.A., Schnabl B. (2011). Enteric dysbiosis associated with a mouse model of alcoholic liver disease. Hepatology.

[62] Ghosh T.S., Shanahan F., O’Toole P.W. (2022). The gut microbiome as a modulator of healthy ageing. Nat. Rev. Gastroenterol. Hepatol..

[63] Tang X., Wang W., Hong G., Duan C., Zhu S., Tian Y., Han C., Qian W., Lin R., Hou X. (2021). Gut microbiota-mediated lysophosphatidylcholine generation promotes colitis in intestinal epithelium-specific Fut2 deficiency. J. Biomed. Sci..

[64] Zhuge A., Li S., Yuan Y., Li B., Li L. (2021). The synergy of dietary supplements *Lactobacillus salivarius* LI01 and *Bifidobacterium longum* TC01 in alleviating liver failure in rats treated with D-galactosamine. Food Funct..

[65] Kim W.G., Kim H.I., Kwon E.K., Han M.J., Kim D.H. (2018). Lactobacillus plantarum LC27 and Bifidobacterium longum LC67 mitigate alcoholic steatosis in mice by inhibiting LPS-mediated NF-κB activation through restoration of the disturbed gut microbiota. Food Funct..

[66] Gurwara S., Dai A., Ajami N.J., Graham D.Y., White D.L., Chen L., Jang A., Chen E., El-Serag H.B., Petrosino J.F. (2020). Alcohol use alters the colonic mucosa-associated gut microbiota in humans. Nutr. Res..

[67] Bjørkhaug S.T., Aanes H., Neupane S.P., Bramness J.G., Malvik S., Henriksen C., Skar V., Medhus A.W., Valeur J. (2019). Characterization of gut microbiota composition and functions in patients with chronic alcohol overconsumption. Gut Microbes.

[68] Leclercq S., Matamoros S., Cani P.D., Neyrinck A.M., Jamar F., Stärkel P., Windey K., Tremaroli V., Bäckhed F., Verbeke K. (2014). Intestinal permeability, gut-bacterial dysbiosis, and behavioral markers of alcohol-dependence severity. Proc. Natl. Acad. Sci. USA.

[69] Du S.Y., Zhang Y.L., Bai R.X., Ai Z.L., Xie B.S., Yang H.Y. (2015). Lutein prevents alcohol-induced liver disease in rats by modulating oxidative stress and inflammation. Int. J. Clin. Exp. Med..

[70] Bardag-Gorce F., Oliva J., Dedes J., Li J., French B.A., French S.W. (2009). Chronic ethanol feeding alters hepatocyte memory which is not altered by acute feeding. Alcohol. Clin. Exp. Res..

[71] Wu X., Fan X., Miyata T., Kim A., Cajigas-Du Ross C.K., Ray S., Huang E., Taiwo M., Arya R., Wu J. (2023). Recent Advances in Understanding of Pathogenesis of Alcohol-Associated Liver Disease. Annu. Rev. Pathol..

[72] Ge N., Liang H., Liu Y., Ma A.G., Han L. (2013). Protective effect of Aplysin on hepatic injury in ethanol-treated rats. Food Chem. Toxicol..

[73] Ma Y., Li R., Liu Y., Liu M., Liang H. (2018). Protective Effect of Aplysin Supplementation on Intestinal Permeability and Microbiota in Rats Treated with Ethanol and Iron. Nutrients.

[74] Ding W.X., Manley S., Ni H.M. (2011). The emerging role of autophagy in alcoholic liver disease. Exp. Biol. Med..

[75] Thomes P.G., Trambly C.S., Fox H.S., Tuma D.J., Donohue T.M. (2015). Acute and Chronic Ethanol Administration Differentially Modulate Hepatic Autophagy and Transcription Factor EB. Alcohol. Clin. Exp. Res..

[76] Chen C., Wang S., Yu L., Mueller J., Fortunato F., Rausch V., Mueller S. (2021). H_2_O_2_-mediated autophagy during ethanol metabolism. Redox Biol..

[77] Chang C.J., Lin J.F., Hsiao C.Y., Chang H.H., Li H.J., Chang H.H., Lee G.A., Hung C.F. (2017). Lutein Induces Autophagy via Beclin-1 Upregulation in IEC-6 Rat Intestinal Epithelial Cells. Am. J. Chin. Med..

[78] Fung F.K., Law B.Y., Lo A.C. (2016). Lutein Attenuates Both Apoptosis and Autophagy upon Cobalt (II) Chloride-Induced Hypoxia in Rat Műller Cells. PLoS ONE.

