# Supplementary material for: Lutein Prevents Liver Injury and Intestinal Barrier Dysfunction in Rats Subjected to Chronic Alcohol Intake
Source: Nutrients. 2023 Feb 28;15(5):1229. doi: 10.3390/nu15051229 (PMC10005241; doi:10.3390/nu15051229)
Supplement: Supplementary file 1 [file nutrients-15-01229-s001.zip › nutrients-2190936-supplementary.pdf]

Supplementary Materials

1 Western Blot

Protein Extraction steps:

1. Homogenate of liver or ileum tissue was prepared using precooled PBS. And the tissue homogenate supernatant was retained.
2. The supernatant was centrifuged at 4 °C, 500 ×g, for 3 min. Subsequently, the supernatant obtained after centrifugation was discarded. The packed cell volume (PCV) was be estimated.
3. For every 20 μl of cell volume, 200 μl of Buffer A (1μl of DTT, 5 μl of 100 mM PMSF, and 5 μl of protease inhibitors were added to each milliliter of Buffer A before use) were added. Then, the centrifuge tube was vortexed vigorously at the maximum speed for 15s, and placed on ice for 10 ~ 15min. The, 11μL cold Buffer B was added, vortexed vigorously at the maximum speed for 5s, and placed on ice for 1min.
4. After vortexed vigorously at maximum speed for 5s, the mixture was centrifuged at 4 °C, 16000×g, for 5min.
5. After centrifugation, the supernatant was transferred into another pre-cooled clean microcentrifuge tube as soon as possible and placed on ice to obtain the cytoplasmic proteins.
6. 100μL of precooled Buffer C (1μl of DTT, 5 μl of 100 mM PMSF, and 5 μl of protease inhibitors were added to each milliliter of Buffer C before use) was added to the centrifuged precipitate (nuclei), vortexed vigorously for 15s at the maximum speed, placed on ice for 40min, and vortexed vigorously for 15 s every 10 min.
7. After centrifugation at 4 °C, 16000×g, for 10min, the supernatant was transferred into a pre-cooled clean microcentrifuge tube as soon as possible to obtain the nucleoproteins.
8. The extracted cytoplasmic and nuclear proteins were quantified by BCA method, packaged and stored at -80℃ to avoid repeated freezing and thawing.

Note: All reagents and instruments used in the experiments need to be precooled.

2 Real-time Quantitative Polymerase Chain Reaction

Real-time PCR lysis amplification curve:

ADH1 gene real-time amplification plot and melt curve

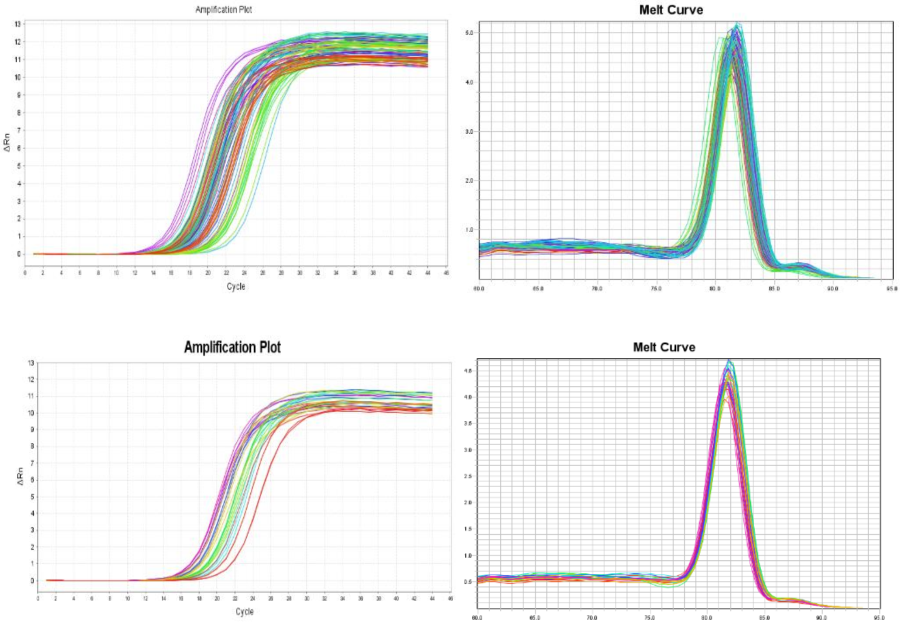

ALDH2 gene real-time amplification plot and melt curve

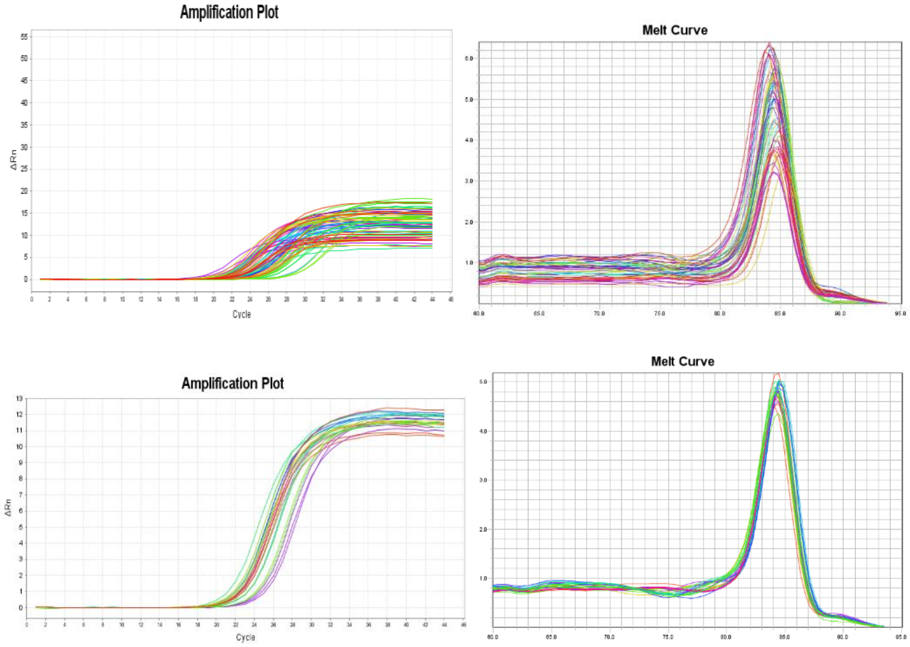

(ADH1 and ALDH2)-β-actin genes real-time amplification plot and melt curve

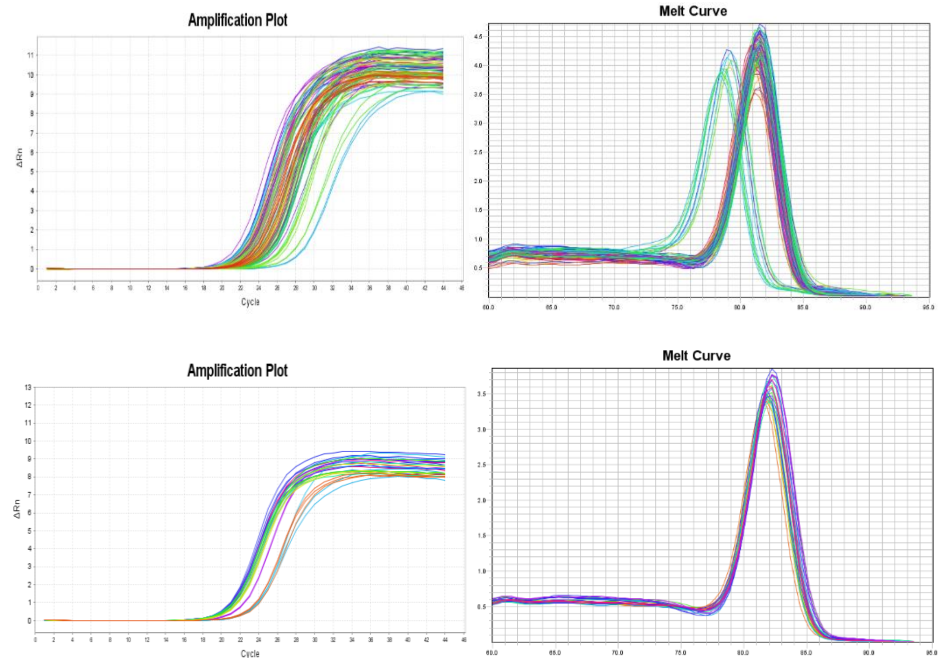

Claudin1 gene real-time amplification plot and melt curve

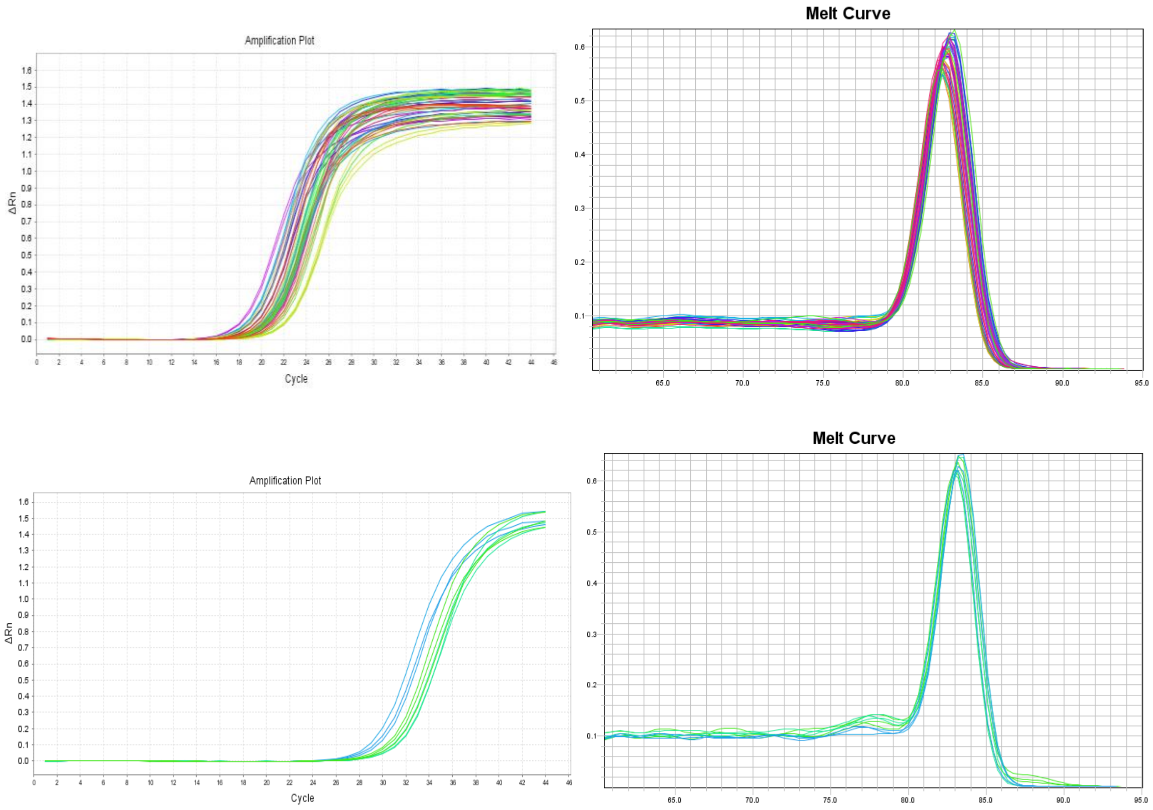

Occludin gene real-time amplification plot and melt curve

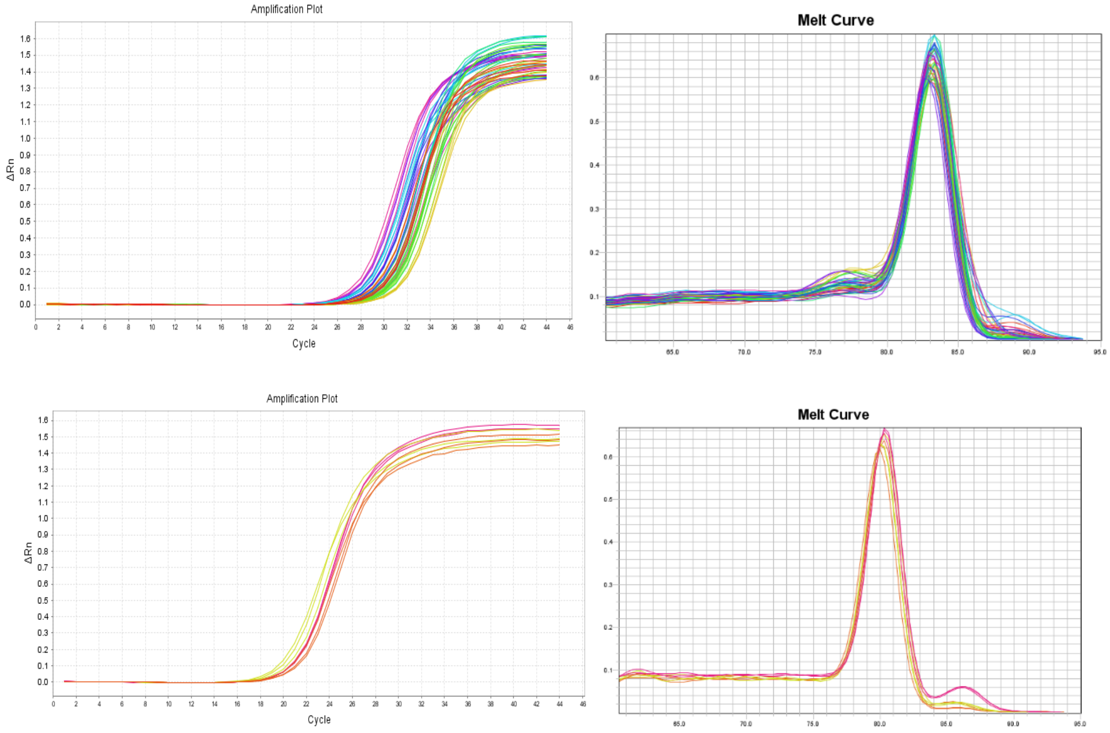

β-actin gene real-time amplification plot and melt curve

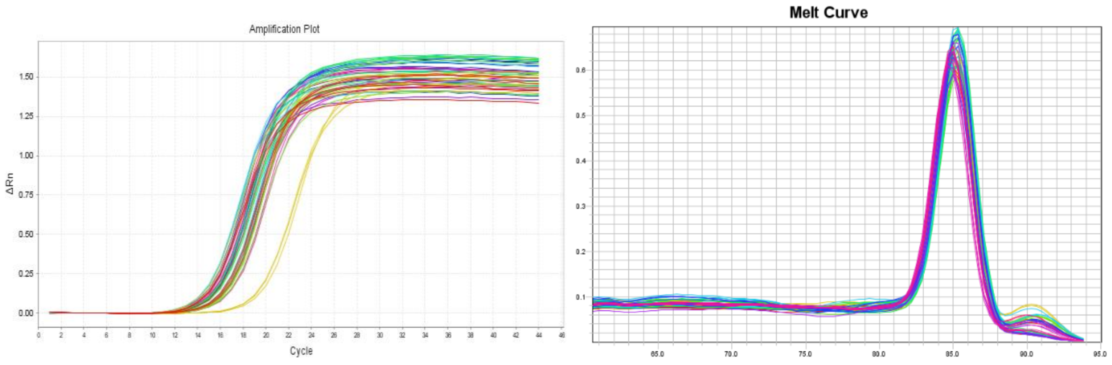

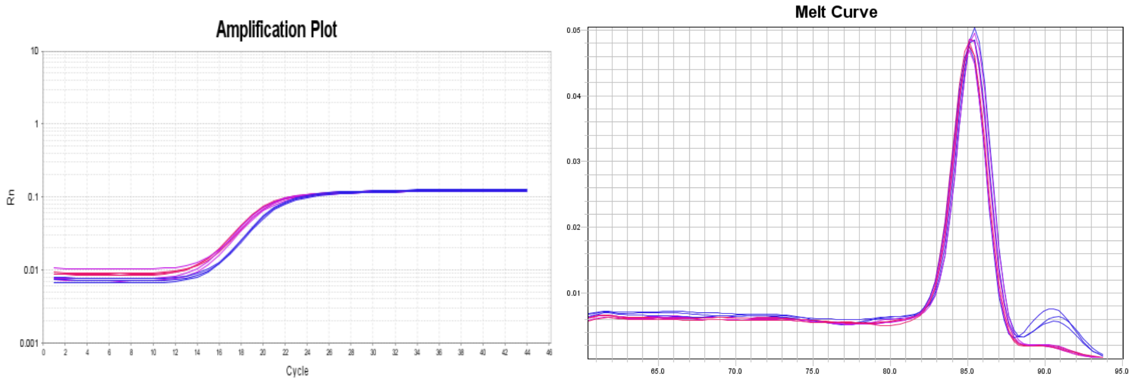

ZO-1 gene real-time amplification plot and melt curve

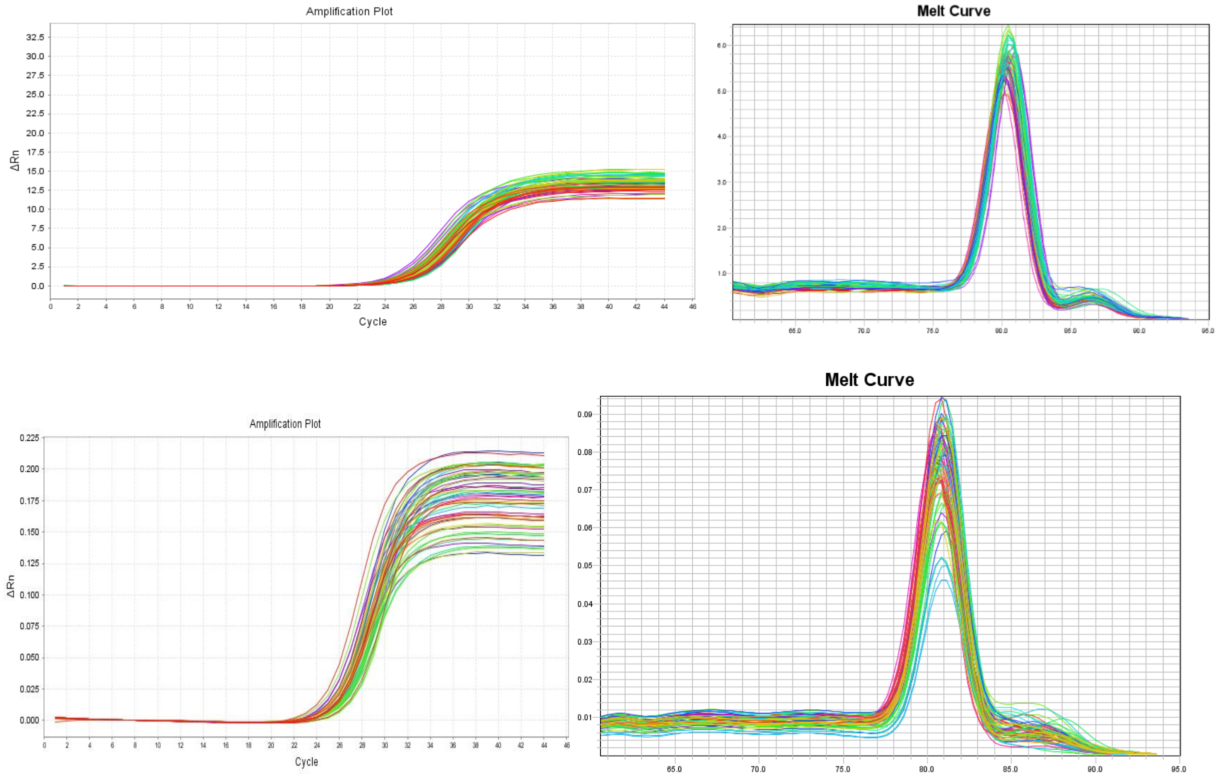

ZO-1-β-actin gene real-time amplification plot and melt curve

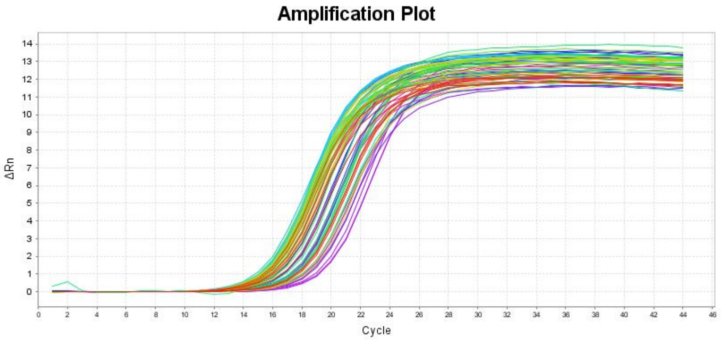
Amplification Plot
ΔRn
Cycle

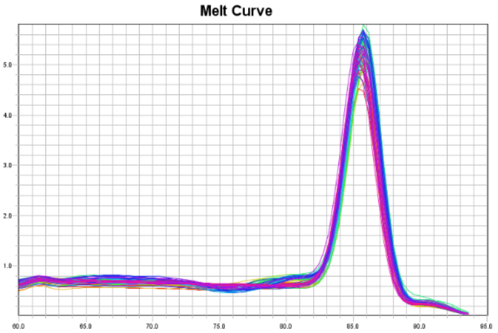
Melt Curve

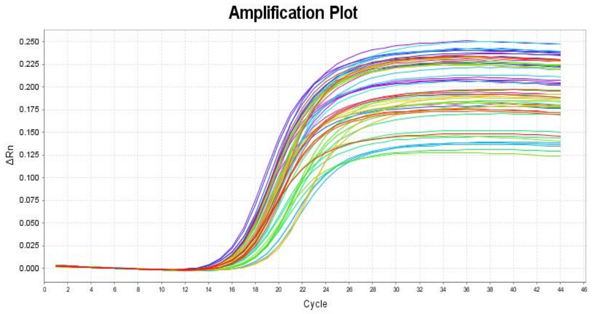
Amplification Plot
ΔRn
Cycle

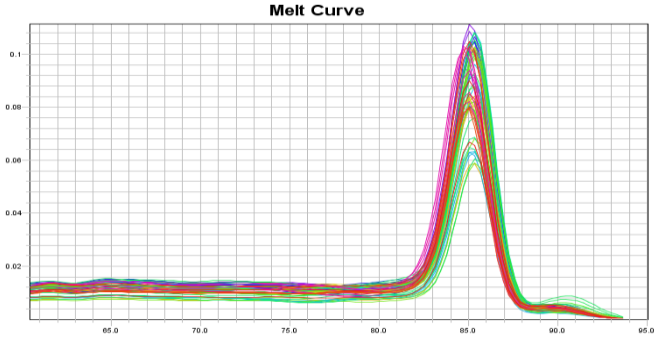
Melt Curve
